# Neuroprotection via Carbon Monoxide Depends on the Circadian Regulation of CD36-Mediated Microglial Erythrophagocytosis in Hemorrhagic Stroke

**DOI:** 10.3390/ijms25031680

**Published:** 2024-01-30

**Authors:** Sandra Kaiser, Luise Henrich, Iva Kiessling, Benedikt Loy, Nils Schallner

**Affiliations:** 1 Department of Anesthesiology & Critical Care Medicine, Medical Center, University of Freiburg, 79106 Freiburg, Germanynils.schallner@uniklinik-freiburg.de (N.S.); 2 Faculty of Medicine, University of Freiburg, 79110 Freiburg, Germany

**Keywords:** circadian rhythm, carbon monoxide, CD36, microglia, phagocytosis, brain hemorrhage

## Abstract

The molecular basis for circadian dependency in stroke due to subarachnoid hemorrhagic stroke (SAH) remains unclear. We reasoned that microglial erythrophagocytosis, crucial for SAH response, follows a circadian pattern involving carbon monoxide (CO) and CD36 surface expression. The microglial BV-2 cell line and primary microglia (PMG) under a clocked medium change were exposed to blood ± CO (250 ppm, 1 h) in vitro. Circadian dependency and the involvement of CD36 were analyzed in PMG isolated from control mice and *CD36^−/−^* mice and by RNA interference targeting *Per-2*. In vivo investigations, including phagocytosis, vasospasm, microglia activation and spatial memory, were conducted in an SAH model using control and *CD36^−/−^* mice at different zeitgeber times (ZT). In vitro, the surface expression of CD36 and its dependency on CO and phagocytosis occurred with changed circadian gene expression. *CD36^−/−^* PMG exhibited altered circadian gene expression, phagocytosis and impaired responsiveness to CO. In vivo, control mice with SAH demonstrated circadian dependency in microglia activation, erythrophagocytosis and CO-mediated protection at ZT2, in contrast to *CD36^−/−^* mice. Our study indicates that circadian rhythmicity modulates microglial activation and subsequent CD36-dependent phagocytosis. CO altered circadian-dependent neuroprotection and CD36 induction, determining the functional outcome in a hemorrhagic stroke model. This study emphasizes how circadian rhythmicity influences neuronal damage after neurovascular events.

## 1. Introduction

There is a noteworthy temporal correlation between the time of day and the frequency and severity of hemorrhagic strokes such as subarachnoid hemorrhage (SAH) [1]. Attributing the likelihood of stroke occurrence solely to external factors, such as physical activity, is an insufficient explanation of this phenomenon [2,3]. Circadian rhythm is inherent to every organism and not only governs sleep–wake cycles but also influences nearly every physiological function [4,5]. Circadian rhythm is primarily generated in the suprachiasmatic nucleus (SCN) of the brain, located in close proximity to the bleeding site during SAH. The molecular basis of circadian rhythmicity consists of the oscillating expression of genes that form negative feedback loops to regulate rhythmicity [6]. This family of “clock genes” consists of several transcription factors, including *CLOCK*, *BMAL-1*, *RevErb-α* and *NPAS-2*, and regulatory proteins, including *Period (Per) 1-3* and *Cryptochrome (Cry) 1-2.* Recent advances in circadian research suggest that almost half of the whole mammalian transcriptome is under circadian control in a highly organ-specific manner [7,8]. Disturbance or deficiency, specifically of Per-1 and Per-2, leads to cardiovascular [9], liver [10] and central nervous system [11] disease. Additionally, the timing of neuronal injury can influence local circadian gene expression profiles and the extent of injury after experimental SAH [12].

We recently identified a novel role for carbon monoxide (CO) generated via heme oxygenase-1 (HO-1) in microglia during SAH: CO mediates the clearance of blood by enhancing microglial erythrophagocytosis [13]. Simultaneously, microglial erythrophagocytosis appears to be dependent on a pathway involving CO-controlled surface expression of the class B scavenger receptor CD36 [14]. CD36 facilitates the recognition and phagocytosis of extravasated erythrocytes [15] and contributes to hematoma resolution after intracranial hemorrhage [16,17,18].

Given the observed circadian correlation observed in SAH in patients, we aimed to further explore our previous findings in the context of circadian rhythmicity. The mechanisms through which CO restores circadian rhythmicity remain unknown [12]. An interesting link exists between circadian control and the HO-1/CO enzyme system: *NPAS-2* [19] and *CLOCK* [20] activity, two transcription factors involved in circadian control via the expression of regulatory *Per* and *Cry* proteins, is modulated by heme binding of CO.

Several questions in this context remain unanswered by the researches’ state of the art and further evaluation is required. This includes determining whether the neuroprotective properties of CO depend on circadian time, how CO-related changes in CD36 expression interact with circadian rhythmicity, and how the changes in central rhythmicity and CD36 expression influence microglial function critical for the response to hemorrhagic injury.

In this comprehensive in vitro and in vivo study, we provide data indicating that circadian rhythm influences microglial function, CD36 surface expression and its responsiveness to CO after SAH. These findings contribute to identifying and further understanding the key determinant of hematoma resolution and neuronal injury following hemorrhagic stroke in the context of circadian timing.

## 2. Results

### 2.1. Circadian Gene Expression Profiles Determine CO-Mediated Induction of CD36 Surface Expression In Vitro

**Figure 1:**
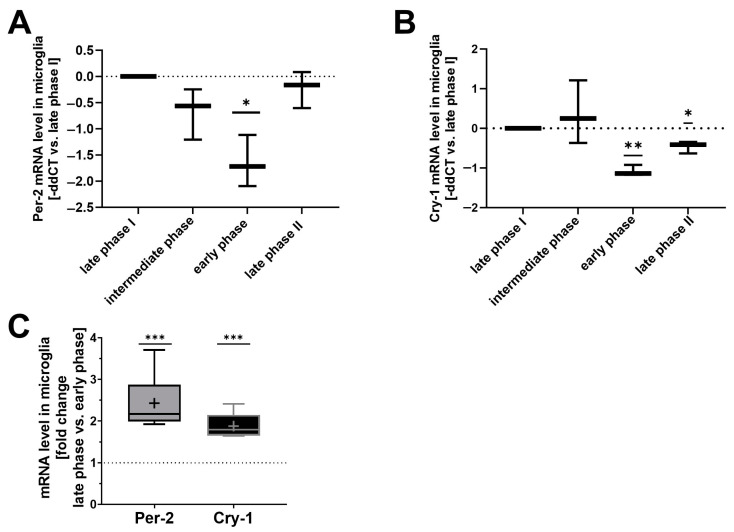
Circadian gene expression profile in cultured primary microglia (PMG) after circadian reset stimulus (**A**,**B**). *Per-2* (**A**) and *Cry-1* (**B**) mRNA levels in cultured PMG after the medium change at 2, 8, 12 or 24 h before harvesting for qPCR analysis. Medium change was used as a circadian reset stimulus resulting in PMG groups conditioned in different circadian phases. Quantification is shown as –ddct (delta delta cycle threshold); *n* = 3 number of independent cell culture preparations; *p* = 0.0287 for *Per-2*; *p* = 0.0052 and *p* = 0.0338 for *Cry-1*; one sample *t*-test versus 0. (**C**) Comparison of *Per-2* and *Cry-1* mRNA levels in cultured PMG. Quantification was performed as fold change late phase versus early phase (phases result from different timings of medium change as circadian rhythm restoration stimuli). *p* = 0.0003 *Per-2*; *p* = 0.0009 *Cry-1*; one sample *t*-test versus 1. *n* = 5–6 number of independent cell culture preparations; box plot: whiskers indicate minimum and maximum, the line in the box marks the median, the “+” in the box shows the position of the mean. Statistically significant values were defined as *p* ≤ 0.05 (* *p* ≤ 0.05; ** *p* ≤ 0.01, *** *p* ≤ 0.001).

Microglial erythrophagocytosis plays a crucial role in hematoma resolution after SAH [15]. Additionally, the time in the day of the occurrence of hemorrhagic stroke and its severity exhibit a robust correlation [1]. We recently elucidated a phagocytosis pathway involving CO-controlled CD36 surface expression on microglia [14]. Therefore, we investigated the circadian dependency of CO treatment and CD36 surface expression in vitro. A distinct oscillating mRNA expression pattern of *Per-2* and *Cry-1* was observed in cultured microglia depending on the timing after the medium change (2 h, 8 h, 12 h, 24 h before cell harvest for downstream analysis) as an entrainment stimulus. This defined an early phase (low expression of *Per-2* and *Cry-1* akin to zeitgeber time 2 (ZT2) of the circadian clock in vivo) [12,21] and a late phase (high expression akin to zeitgeber time 12 (ZT12) in vivo), corresponding to the normal 24 h circadian oscillation in vivo (Figure 1A–C).

**Figure 2:**
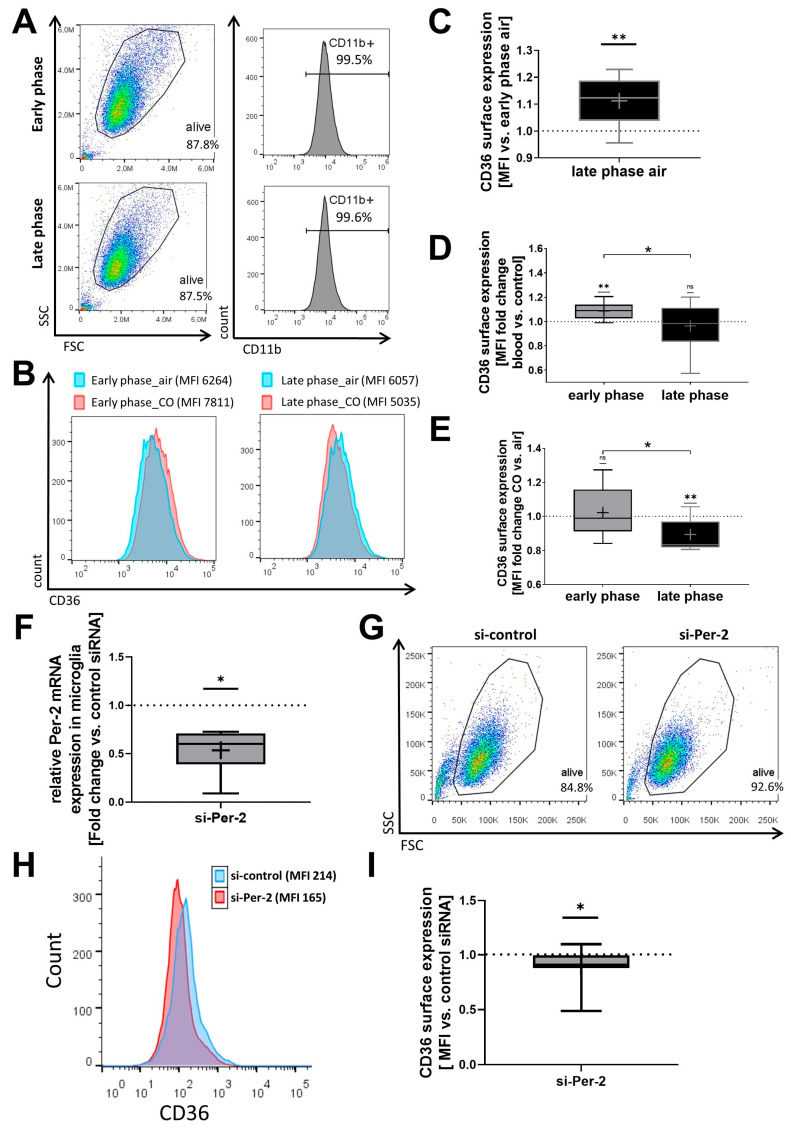
Circadian gene expression profile alters CD36 surface expression and its induction by carbon monoxide (CO). CD36 surface expression in a microglia cell line (BV-2) measured by flow cytometry analysis. Early and late phases result from different timings of medium change as circadian rhythm restoration stimuli. (**A**) Representative flow cytometry blots and surface staining histograms (CD11b was used as microglia marker). (**B**) Representative flow cytometry histograms for CD36 surface staining (MFI: mean fluorescent intensity). (**C**) MFI quantified as fold change late phase vs. early phase. *p* = 0.0078. (**D**) MFI quantified as fold change after blood exposure (1 h before cell harvest) versus control (no blood). *p* = 0.0378 late phase vs. early phase; *p* = 0.0055 one sample *t*-test early phase versus 1. (**E**) MFI quantified as fold change CO treatment (250 ppm, 1 h before harvest) versus air (as control). *p* = 0.0408 late phase vs. early phase; *p* = 0.0084 one sample *t*-test late phase versus 1. *n* = 9 number of independent cell culture preparations (**C**–**E**). (**F**) siRNA knockdown of Per-2 in BV-2 cells versus negative control siRNA demonstrated by qPCR analysis. *p* = 0.0313 one sample Wilcoxon test (presented as 2-exponent-minus-delta-delta-ct (normalization group with control siRNA = 1); statistic performed on delta-delta-ct values: early phase versus 0). *n* = 7 number of independent cell culture preparations. (**G**,**H**) Representative flow cytometry of forward and side scatter (**G**) and histograms for CD36 surface staining (MFI: mean fluorescent intensity) (**H**) of siRNA-treated BV-2 cells. (**I**) MFI quantification of CD36 staining as fold change versus si-control. *p* = 0.0489 one sample *t*-test si-*Per-2* versus 1. *n* = 13 number of independent cell culture preparations; box plot: whiskers indicate minimum and maximum, the line in the box marks the median, the “+” in the box shows the position of the mean. Statistically significant values were defined as *p* ≤ 0.05 (* *p* ≤ 0.05; ** *p* ≤ 0.01).

Concomitantly, CD36 surface expression varied in a circadian manner: microglia exhibited less baseline CD36 surface expression during the phase of low circadian gene expression (early phase) compared to microglia with high expression levels (late phase) (Figure 2A–C). In the early phase, the presence of blood increased CD36 surface expression, while no such blood-related increase was observed during the phase of high circadian gene expression (Figure 2D).

Microglia in the early or late circadian phase, corresponding to either low or high *Per-2/Cry-1* expression levels, demonstrated a different susceptibility towards CO-mediated changes in CD36 surface expression (Figure 2E). Additionally, siRNA experiments with partial Per-2 knockdown in a microglia cell line further demonstrated that cells with lower *Per-2* expression exhibit reduced CD36 surface expression (Figure 2F,I). These data indicate that circadian rhythmicity determines CD36 surface expression and the related effects of CO treatment on CD36 in cultured microglia.

### 2.2. The Phagocytic Function of Primary Microglia (PMG) and Their Responsiveness to Exogenous CO Is Circadian-Dependent In Vitro

**Figure 3:**
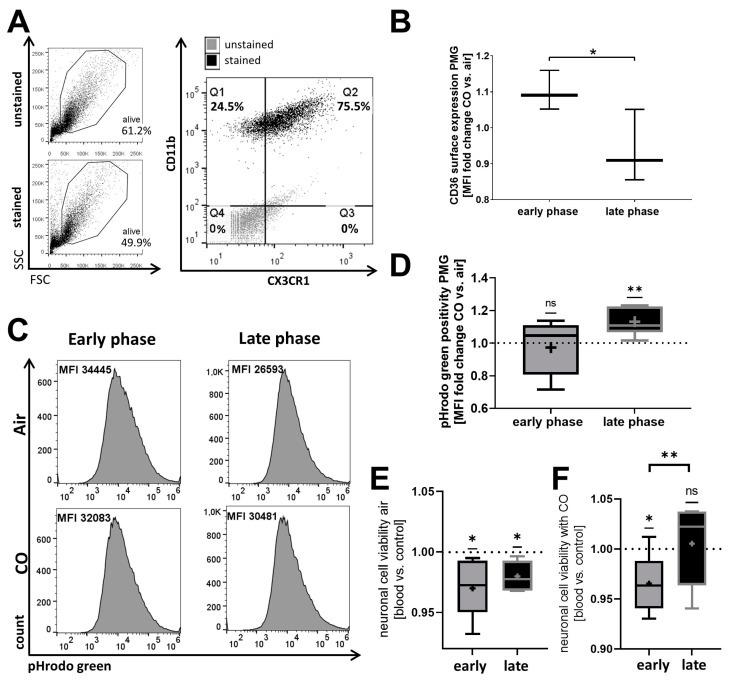
Circadian rhythmicity combined with carbon monoxide (CO) influences phagocytosis function of primary microglia (PMG) and its impact on neuronal viability. (**A**) Flow cytometry of forward versus side scatter and CD11b with CX3CR1 surface expression in cultured PMG. Overlay of dot blots of stained and unstained cells of the corresponding alive gates. (**B**) CD36 surface expression in cultured control PMG. Mean fluorescent intensity (MFI) quantified as fold change CO treatment (250 ppm, 0.5 h before harvest) versus air (as control). Early and late phases result from different timings of medium change as circadian rhythm restoration stimuli. *n* = 3 number of independent cell culture preparations; *p* = 0.0267. (**C**,**D**) Erythrophagocytosis in cultured control PMG was analyzed by flow cytometry after 1 h of exposure to blood fluorescently labeled with pHrodo green. (**C**) Representative flow cytometry histograms for pHrodo green positive cells (MFI: mean fluorescent intensity). (**D**) Quantification was performed as fold change for CO treatment (250 ppm, 1 h) versus air; *n* = 7 number of independent cell culture preparations; *p* = 0.0059 one sample *t*-test late phase versus 1. (**E**,**F**) Co-cultivation of the neuronal cell line HT22 with PMGs 12 h stimulated with blood. Medium change as circadian reset stimulus was performed 2 or 12 h (indicated as early and late phase PMG) before the addition of blood and co-culture start. Viability assay is shown as fold change versus without blood stimulus either exposed to air as control (**E**) or treated with CO (250 ppm, 1 h before cell harvest for downstream analysis) (**F**). *p* = 0.0116 early phase and *p* = 0.0324 late phase one sample *t*-test versus 1 (**E**). *p* = 0.0318 one sample *t*-test early phase versus 1. *p* = 0.0094 early versus late phase *t*-test (**F**). *n* = 6 number of independent cell culture preparations; box plot: whiskers indicate minimum and maximum, the line in the box marks the median, the “+” in the box shows the position of the mean. Statistically significant values were defined as *p* ≤ 0.05 (* *p* ≤ 0.05; ** *p* ≤ 0.01).

To further validate the circadian dependency of erythrophagocytosis, murine PMG were examined for their phagocytic function in vitro. Cultured PMG expressed CD11b and CX3CR1, indicative of their microglial character (Figure 3A). Consistent with earlier findings in a microglial cell line (Figure 2E), circadian rhythm altered the impact of CO on CD36 surface expression in PMG (Figure 3B). Since CD36 promotes phagocytosis of erythrocytes [15], the circadian influence on phagocytosis was further investigated. The addition of blood to microglia at the late circadian phase resulted in a CO-dependent increase in erythrophagocytosis. No such CO-related effect was observed in microglia during times of low circadian gene expression in the early phase of the circadian cycle (Figure 3C,D). Observing this circadian influence on microglial erythrophagocytosis, we further explored whether this also resulted in concomitant changes in neuronal viability after blood exposure at different circadian times using in vitro co-culture experiments. Co-cultivation of blood-stimulated microglia and neuronal cells resulted in a reduction in neuronal cell viability, irrespective of the PMGs’ circadian phase (Figure 3E). In contrast, blood stimulation of microglia during the late phase, characterized by higher expression of *Per-2* and *Cry-1*, did not reduce neuronal viability with CO treatment. Conversely, during the early phase, blood induction reduced neuronal viability despite CO exposure (Figure 3F), further indicating a circadian dependency of CO-mediated erythrophagocytosis and the inflammatory state in microglia, indirectly influencing neuronal cell viability.

### 2.3. CD36^−/−^ PMG Lack Circadian Rhythmicity and Responsiveness to CO Exposure In Vitro

**Figure 4:**
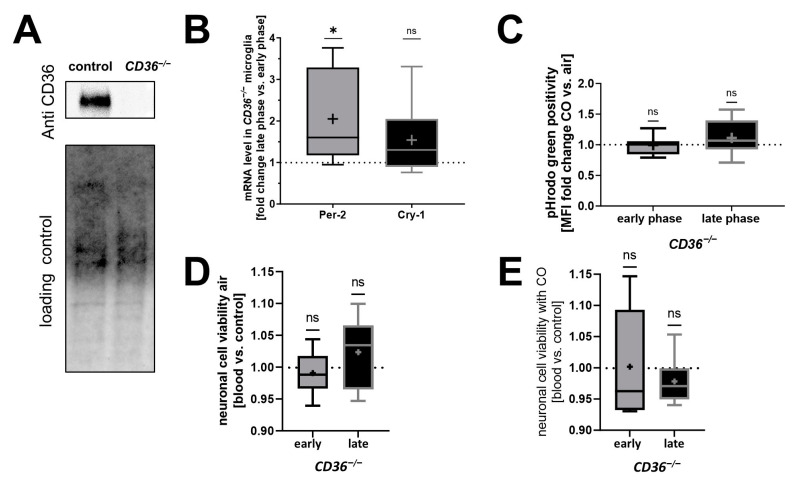
Circadian rhythmicity and carbon monoxide (CO) exposure in CD36-deficient primary microglia (PMG) in vitro. (**A**) Western blotting showing CD36 protein level in cultured PMG of control mice compared to no CD36 expression in *CD36^−/−^* mice. The lower panel displays the corresponding control of the loaded protein, serving as a reference in the comparison. Thus, stainless UV-light-based imaging of the total protein was used. (**B**) Comparison of *Per-2* and *Cry-1* mRNA levels in cultured *CD36^−/−^* PMG. Quantification was performed as fold change late phase versus early phase. *n* = 6 number of independent cell culture preparations; *p* = 0.0440 *Per-2* one sample *t*-test versus 1. (**C**) Erythrophagocytosis in cultured *CD36^−/−^* PMG analyzed by flow cytometry after 1 h exposure to blood fluorescently labeled with pHrodo green. Quantification was performed as fold change for CO treatment (250 ppm, 1 h) versus air (as control); *n* = 7 number of independent cell culture preparations. (**D**,**E**) Co-cultivation of the neuronal cell line HT22 with *CD36^−/−^* PMGs 12 h stimulated with blood. The medium change as circadian reset stimulus was performed 2 or 12 h (indicated as early and late phase PMG) before the addition of blood and co-culture start. Viability assay is shown as fold change versus without blood stimulus either exposed to air as control (**D**) or treated with CO (250 ppm, 1 h before cell harvest for downstream analysis) (**E**). *p* = n.s. (non-significant) one sample *t*-test versus 1. *n* = 6 number of independent cell culture preparations; box plot: whiskers indicate minimum and maximum, the line in the box marks the median, the “+” in the box shows the position of the mean. Statistically significant values were defined as *p* ≤ 0.05 (* *p* ≤ 0.05).

To further study the role of CD36 within the context of circadian rhythm and CO treatment, we examined CD36 knockout (KO) mice and isolated microglia from these animals (Figure 4A). Akin to control microglia, *Per-2* expression in CD36 KO microglia was increased during the late circadian phase compared to the early phase. Unlike control microglia (Figure 1C), microglia lacking CD36 showed no variation in *Cry-1* expression at different circadian times (Figure 4B), indicating a circadian influence on *Cry-1* expression dependent on CD36. Additionally, microglia lacking CD36 displayed no CO-dependent increase in phagocytosis during the late phase with high circadian gene expression (Figure 4C), in contrast to control microglia (Figure 3C,D). CD36-deficient PMGs did not affect the viability of co-cultured neurons after blood exposure in both the early and late phase (Figure 4D). In addition, exogenous CO did not impact the viability of neurons co-cultured with CD36-deficient microglia, regardless of the circadian phase (Figure 4E).

In summary, *CD36^−/−^* microglia exhibited altered circadian rhythmicity along with an absence of responsiveness to CO exposure concerning erythrophagocytosis.

### 2.4. Erythrophagocytosis and Neuroinflammation Depend on the Circadian Timing of Injury and Expression of CD36 In Vivo

**Figure 5:**
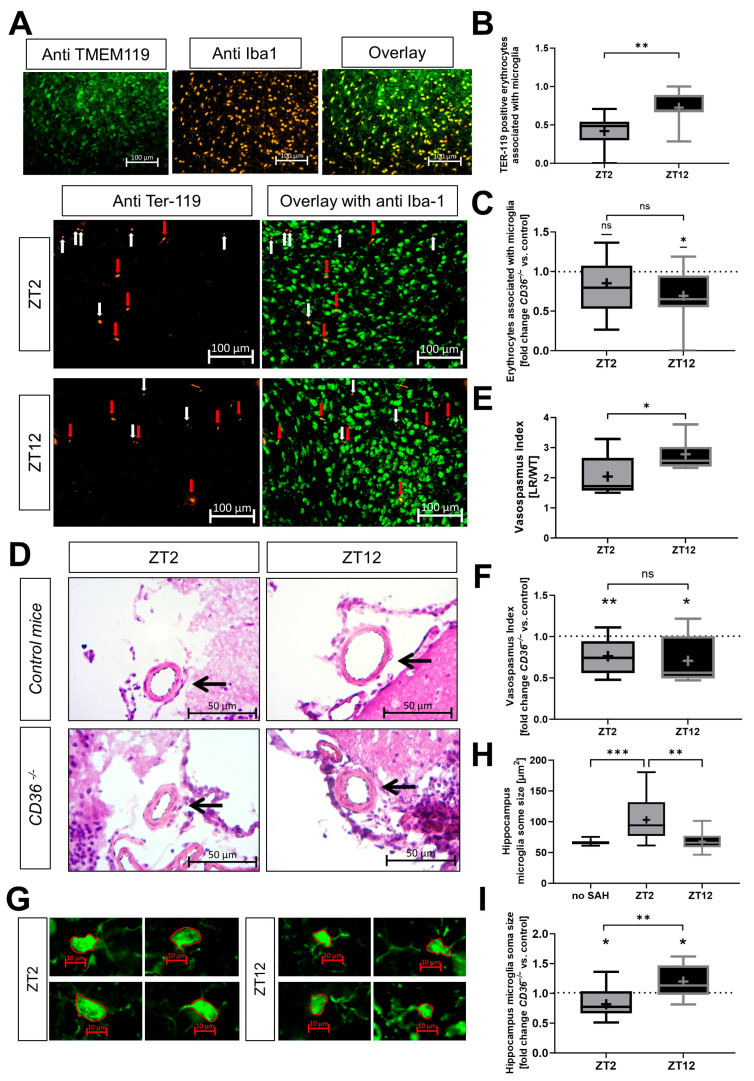
Influence of circadian timing of subarachnoid hemorrhage (SAH) induction on inflammatory response in control and *CD36^−/−^* mice. (**A**–**C**) Analysis of erythrophagocytosis in vivo by immunofluorescent staining at the brain base (site of blood injection) in control and *CD36^−/−^* mice 7 days post-SAH induced at zeitgeber time (ZT) 2 or 12. (**A**) Representative microglia marker staining with antibodies against TMEM119 and Iba1 and their co-localization are shown as an overlay for the fluorescent signal. For phagocytosis analysis, co-localization of immunofluorescent-stained Ter-119-positive erythrocytes and Iba-1-stained microglia was analyzed (shown representative). Scale bars represent 100 µm. White arrows mark the Ter-119 signal alone. Red arrows mark the co-localization of Ter-119 and Iba-1 signals. (**B**) Quantification of microglia-associated TER-119 signals. The calculation was performed by counting co-localized TER-119-positive erythrocytes with Iba-1-positive microglia divided by total counts of TER-119 signals. Control mice; *n* = 9–12 analyzed images per group; *p* = 0.0094 ZT2 vs. ZT12. (**C**) Fold change in *CD36^−/−^* mice versus control mice; *n* = 9–12 analyzed images per group; *p* = 0.0196 ZT12 one sample *t*-test versus 1. (**D**–**F**) Analysis of vasospasm on Hematoxylin/Eosin-stained cross-sections of the middle cerebral artery (MCA) at the base of the brain of control and *CD36^−/−^* mice 7 days post-SAH induced at ZT2 or ZT12. (**D**) Representative Hematoxylin/Eosin-stained cross-sections of the MCA at the base of the brain. Arrows mark the analyzed vessels. Scale bars represent 50 µm. (**E**) Quantification of the vasospasm index (lumen radius (LR)/wall thickness (WT)) in the MCA. Control mice; *n* = 3–9 analyzed images per group; *p* = 0.0263 ZT2 vs. ZT12. (**F**) Fold change in *CD36^−/−^* mice versus control mice; *n* = 9–12 analyzed images per group; *p* = 0.0034 ZT2 and *p* = 0.0154 ZT12 one sample *t*-test versus 1. (**G**–**I**) Coronal brain sections were fluorescently stained for Iba-1 in the hippocampus of control and *CD36^−/−^* mice 7 days post-SAH induced at ZT2 or ZT12. (**G**) Representative microglia in coronal brain sections fluorescently stained for Iba-1. Red borders mark measured cell soma area. Scale bars represent 10 µm. (**H**) Quantification of microglia activation was performed using the morphological criteria microglia soma size. *n* = 12 analyzed images per group; ordinary one-way ANOVA: no SAH control mice vs. ZT2 *p* = 0.0006 and ZT2 vs. ZT12 *p* = 0.0011. (**I**) Fold change *CD36^−/−^* mice versus control mice; *n* = 9–12 analyzed images per group; *p* = 0.0023 ZT2 versus ZT12; *p* = 0.0394 ZT2 and *p* = 0.0266 ZT12 one sample *t*-test versus 1; box plot: whiskers indicate minimum and maximum, the line in the box marks the median, the “+” in the box shows the position of the mean. Statistically significant values were defined as *p* ≤ 0.05 (* *p* ≤ 0.05; ** *p* ≤ 0.01, *** *p* ≤ 0.001).

To further substantiate the in vitro results, additional in vivo analyses were conducted in *CD36^−/−^* and control mice to elucidate the relationship between the circadian rhythm and CD36 expression in hemorrhagic stroke. Microglia expressing Iba-1 along with TMEM119 were examined in mouse brain sections to confirm the specificity of the subsequent analyses (Figure 5A). To assess phagocytosis activity in vivo, Ter119-positive erythrocytes binding to microglia after hemorrhage were investigated. Control mice exhibited higher phagocytic activity after hemorrhage at the late circadian phase with high expression of circadian control genes (ZT12) compared to the early phase of the circadian cycle with corresponding low expression levels (ZT2) (Figure 5A,B). Furthermore, CD36 knockout mice at ZT12 displayed reduced phagocytosis activity compared to control mice (Figure 5C). Additionally, ZT2 control mice showed enhanced vasospasm versus ZT12 (Figure 5D,E). Higher vasospasm was observed in mice lacking CD36, independent of the circadian timing of SAH (Figure 5F). The morphology of microglia in control mice shifted towards a stronger inflammatory response at ZT2 compared to ZT12 and control mice without SAH induction (Figure 5G,H). In contrast to these findings, *CD36^−/−^* mice displayed reduced microglial activation at ZT2, while higher activation was observed after hemorrhage at ZT12 (Figure 5I). In summary, the induction of hemorrhagic injury at ZT2 leads to less phagocytosis, higher vasospasm and increased microglia activation compared to ZT12. The genotypic effect caused by the loss of CD36 results in alterations in the inflammatory response and loss of the circadian dependency of the described effects.

### 2.5. The Protective Effect of CO Depends on the Circadian Timing of Injury and Treatment

**Figure 6:**
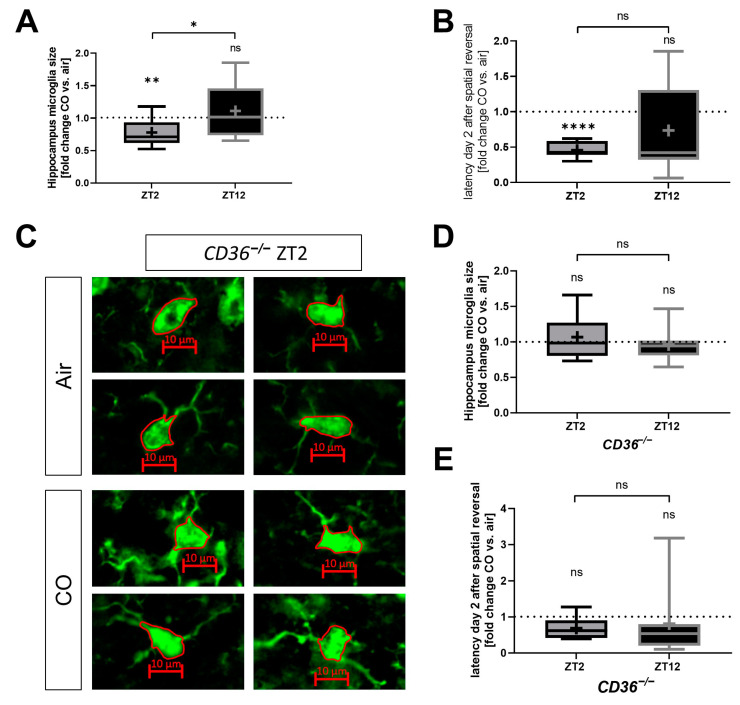
Circadian dependency of the protective effect of carbon monoxide (CO) in vivo after subarachnoid hemorrhage (SAH) in control and *CD36^−/−^* mice. (**A**) Coronal brain sections were fluorescently stained for Iba-1 in the hippocampus of mice with CO treatment (1 h, 250 ppm daily) 7 days after subarachnoid hemorrhage (SAH). ZT2 and ZT12 indicate the zeitgeber timing of the SAH induction and thus of the occurrence of brain bleeding. Quantification of microglia activation was performed using the morphological criteria microglia soma size and is expressed as fold change CO versus air treatment. *p* = 0.0161 zeitgeber time 2 (ZT2) versus zeitgeber time 12 (ZT12); *p* = 0.0035 one sample *t*-test ZT2 versus 1; *n* = 9–12 analyzed images per group. (**B**) Relative latency times in the Barnes maze spatial memory paradigm (for functional outcome analysis) on day two after the spatial reversal (6 days after SAH) in control mice with CO treatment expressed as fold change versus air; *p* ≤ 0.0001 one sample *t*-test ZT2 versus 1; *n* = 7 animals per group. (**C**,**D**) Coronal brain sections were fluorescently stained for Iba-1 in the hippocampus of *CD36^−/−^* mice with CO treatment (1 h, 250 ppm daily) 7 days after SAH and analyzed as fold change versus air control. (**C**) Representative microglia in coronal brain sections of *CD36^−/−^* mice fluorescently stained for Iba-1. Red borders mark measured areas of cell somata. Scale bars represent 10 µm. (**D**) Quantification of microglia activation was performed using the morphological criteria microglia soma size. *p* = n.s. (non-significant) one sample *t*-test versus 1 and unpaired *t*-test; *n* = 12–15 analyzed images per group. (**E**) Relative latency times on day two after spatial inversion (6 days after SAH) in *CD36^−/−^* mice with CO treatment expressed as fold change versus air control; *p* = n.s. one sample Wilcoxon test versus 1 and Mann–Whitney test; *n* = 6 (zeitgeber time 2 (ZT2)) and 8 (zeitgeber time 12 (ZT12)) animals per group; box plot: whiskers indicate minimum and maximum, the line in the box marks the median, the “+” in the box shows the position of the mean. Statistically significant values were defined as *p* ≤ 0.05 (* *p* ≤ 0.05; ** *p* ≤ 0.01, **** *p* ≤ 0.0001).

Furthermore, we investigated whether the circadian timing of injury influences the neuroprotective effects of CO after hemorrhagic stroke in vivo. A significant reduction in microglia activation was observed with CO treatment in mice with SAH during the early phase of the circadian cycle (ZT2). No such CO effect was detected in mice being injured at the late phase of the circadian cycle (ZT12) (Figure 6A). Accordingly, CO enhanced the functional outcome regarding spatial memory function in Barnes maze experiments only in ZT2, not in ZT12 mice (Figure 6B). In summary, a circadian dependency was identified concerning CO-related neuroprotection after hemorrhagic injury.

### 2.6. CO Does Not Protect CD36^−/−^ Mice from Neuronal Injury Irrespective of Circadian Timing

In vitro experiments demonstrated a lack of responsiveness to CO exposure in CD36-deficient microglia (Figure 4). Additionally, CD36 KO mice exhibited an altered response to SAH induction (Figure 5). Therefore, CO exposure was further investigated in vivo in CD36-deficient mice after hemorrhagic injury. CO treatment of *CD36^−/−^* mice did not alter microglia activation independent of circadian timing of the SAH induction (Figure 6C–E) in contrast to control mice (Figure 6A). Additionally, no CO effect could be observed in *CD36^−/−^* mice in the functional outcome in the Barnes maze experiments, neither at ZT2 (like control mice, Figure 6B) nor ZT12 after SAH induction (Figure 6E). These data suggest a crucial role for CD36 in inflammation, erythrophagocytosis and functional outcome after hemorrhagic injury and point towards a CD36 dependency of exogenous CO treatment.

## 3. Discussion

The results of this study demonstrate that microglial activation, phagocytic capacity and injury response post-SAH are modulated by endogenous circadian rhythmicity. The impact of the gaseous molecule CO on the inflammatory response and neuroprotection after cerebral hemorrhage, as shown before [14,22,23], was influenced by the circadian timing of injury and treatment. Additional analysis revealed that the circadian gene expression profile in microglia impacts CO-mediated CD36 surface expression and thus microglial phagocytosis of erythrocytes, as CD36 surface expression is reduced after the knockdown of Per-2. Studying a *CD36^−/−^* KO model further verified that microglia lacking CD36 expression are deficient in circadian rhythmicity and responsiveness to CO exposure in vitro with respect to erythrophagocytosis and influence on neuronal viability. The lack of circadian dependency regarding the inflammatory response and functional outcome after hemorrhage was further demonstrated in *CD36^−/−^* mice in vivo to support the previous notion that circadian rhythmicity determines protective effects related to CD36 and CO.

In humans, an interesting correlation exists between the likelihood of hemorrhagic cerebral injuries such as SAH and the time of day when the injury occurs, with the greatest likelihood in the early morning [1,2]. This might be explained by the fact that our internal circadian rhythm determines nearly every physiological function [4]. Circadian rhythm is primarily generated by the oscillating expression of genes that form a negative feedback loop [6,24]. This family of “circadian rhythm genes” consists of transcription factors including CLOCK, BMAL-1, RevErb-α and NPAS-2 and regulatory proteins including Period (Per) 1-3 and Cryptochrome (Cry) 1-2. Deficiency in these genes, particularly in the regulatory Period genes *Per-1* and *Per-2*, leads to systemic disease including the central nervous system [9,11]. Meanwhile, the HO-1/CO enzyme system is linked to circadian regulation: the transcriptional activity of NPAS-2 [19] and CLOCK [20] is CO-dependent via a heme-based gas sensor mechanism. Additionally, we previously demonstrated that hemorrhagic brain injury leads to disturbances in circadian rhythmicity in mice [12] and humans [25]. However, the exact molecular mechanisms of how circadian rhythmicity influences the extent of neuronal injury remain unclear.

Hemorrhagic cerebral injuries such as SAH lead to neuronal injury. The destruction of brain tissue through cell death can be caused by apoptosis, but it is not the only cause of cell death as SAH induces a complex process with necrosis, excitotoxicity, oxidative stress and inflammation taking place simultaneously [26,27]. Based on our SAH model, we published data concerning apoptosis and inflammation post-SAH in mice [12,14]. Hemorrhagic neuronal brain injury post-SAH via heme-induced cerebral inflammation can be accompanied by a disruption of the blood–brain barrier [28,29,30]. Post-SAH, HO-1 is mainly upregulated in microglia during the cerebral immune response [31,32,33,34]. Heme containing erythrocytes can pass through into the adjacent brain tissue during SAH and interact with phagocytizing, heme oxygenase (HO)-containing microglia. The elimination of heme usually occurs through the HO enzymes that degrade heme into biliverdin, iron and CO. Induction of the inducible HO-1 isoform exerts strong cytoprotective effects in numerous disease models, including the central nervous system, via the release of CO [35,36,37,38,39,40]. We recently identified a novel role for CO released via microglial HO-1 activity in response to hemorrhagic stroke in that microglial CO production not only mediates the clearance of blood but also regulates erythrophagocytosis via the expression of the scavenger receptor CD36 [13,14]. Due to the circadian correlation observed in SAH patients, it is of interest to understand the mechanism of the circadian dependency of the neuroprotective effect via CO. SAH patients exhibit a disturbance in their circadian rhythmicity, which correlates with unfavorable outcomes [12]. It is established that CO can restore circadian rhythmicity, presenting a potential therapeutic usage [12]. Published studies have indicated that the circadian transcription factors NPAS and CLOCK can bind to heme, leading to the modulation of their DNA binding. Moreover, heme-bound NPAS and CLOCK can form complexes with CO, thereby inhibiting DNA binding [19,20]. In accordance with the findings in this study, microglial CD36 determines phagocytosis after intracerebral hemorrhage [16] and other cerebral injury modalities [17,18,41,42,43]. Additionally, a deficiency of CD36 has been linked to increased neuronal injury [16], while at the same time, its functional or genetic absence impairs microglia and macrophage function [17,41,44]. However, circadian dependency of the mechanisms involving CD36 and the related functions of erythrophagocytosis and heme removal have not been revealed. The canonical biological function of the scavenger receptor CD36 must be seen in fat metabolism [45,46]. Interestingly, its metabolic function has been linked to the immune response, providing a link between metabolism and immunity [47]. CD36 expression is described as dysregulated after experimental circadian disruption in mice [48]. As an additional link to circadian rhythm, data from experimental circadian dysregulation studies suggest that circadian rhythmicity and the function of CD36 are closely related and determine the extent of metabolic disease [49,50]. A molecular mechanism outlining the regulation of CD36 expression through hypoxia-inducible factor 1α (Hifα) has been published [49]. Hifα, a subunit of the transcription factor hypoxia-inducible factor-1 (HIF-1), regulates the response to hypoxia and plays a crucial role in ischemic injury, which also takes place in the brain following SAH [51,52]. The circadian gene *CLOCK* can inhibit Hifα expression, resulting in lower Hifα expression with a subsequent reduction in CD36 expression. This relationship between CD36 and circadian regulation aligns with our data. We observed elevated CD36 surface expression in the late phase, equivalent to the circadian period with lower *CLOCK* and higher *Per-2* expression levels. Additionally, Per-2 knockdown resulted in a reduction in CD36 surface expression. When considered alongside the CO modulation of CLOCK DNA-binding capacity, these findings provide insights into a potential mechanism underlying the circadian dependency of the neuroprotective effect of CO in the context of CD36-mediated phagocytosis in SAH.

Other studies have established a close relationship between circadian gene expression profiles in the heart and cardiac expression of metabolic key regulators such as CD36 [53]. In our experiments, CD36 knockout mice displayed disturbed circadian regulation, providing evidence for the close relation between circadian rhythmicity and the functional relevance of CD36, not only via circadian regulation of CD36 but also vice versa. Here, we present a novel pathway for how CD36 influences circadian rhythmicity and its related changes in injury response.

The main limitation of this study is that the majority of functional studies were conducted in vitro. However, due to the complexity of analyzing the temporal interactions between the pathways, PMG had to be utilized. In vitro studies on the influence of circadian rhythm have been shown to be feasible with robust expression patterns concerning genes relevant to circadian control [54]. While neuronal cells of the SCN maintain circadian gene expression rhythmicity ex vivo, other cultivated cells lose their circadian gene expression rhythmicity. Nevertheless, circadian rhythmicity can be restored with sufficient stimuli such as medium change and other modalities [54]. We provided additional evidence in our cell model that circadian oscillation of gene expression can be sustained in vitro for at least 24 h. Timing the medium change of cultured microglia allowed us to study interventions and treatments at different time points in the circadian rhythm, as demonstrated by distinct gene expression patterns of *Per-2* and *Cry-1*, which would not have been possible in pure in vivo studies.

Further limitations concern our in vivo studies. We modelled the complex occurrence of SAH by injecting a certain amount of blood into a stereotactically defined region in the brain base. While this approach enabled us to study and compare distinct mechanisms, it clearly lacks the complexity of the clinical presentation of SAH observed in diverse patients. Regarding SHAM mice, unfortunately, it is not practical to compare erythrophagocytosis in mice with no hematoma containing erythrocytes in the brain (SHAM) versus animals in which SAH has occurred, as this analysis would have no biological significance in the context of this study. Therefore, we focused on relative comparisons between genotypes and over time for this endpoint.

The mentioned CO dose has been established in animal experiments and even in human studies, resulting in non-toxic carboxyhemoglobin levels while providing physiological effects [55,56]. There is an ongoing debate about whether the amounts used exogenously truly reflect endogenous levels produced via HO-1 induction. However, the physiological effects of endogenous and exogenous CO are comparable concerning their influence on cellular respiration [57] and their effect on microglia function, as exogenous application can compensate for endogenous lack in certain scenarios [13].

In conclusion, our data demonstrate that injury response post-SAH, microglial activation and CD36-mediated phagocytosis via the gaseous molecule CO are dependent on the circadian timing of injury and treatment. These new insights into the molecular and temporal patterns in hemorrhagic brain injury might improve the understanding of the complex molecular mechanisms involved in neuroprotection and circadian dependency. In the future, CO might prove to be a beneficial adjunct to stroke therapy. Understanding how the central and peripheral clocks influence the injury response may offer critical clues as to how and, perhaps more importantly, when to intervene with therapy.

## 4. Materials and Methods

### 4.1. Animal Strains, Isolsated Microglia Cells and BV-2 Cellline in Culture

*CD36*^−/−^ (#019006; RRID:IMSR_JAX:019006) mice were obtained from the Jackson Laboratory, Bar Harbor, ME, USA. *Hmox1^fl/fl^* mice (Riken Bio Resource Center, 3-1-1 Koyadai, Tsukuba, Ibaraki, Japan, RBRC03163; RRID:IMSR_RBRC03163) without Cre expression served as the control animals, exhibiting normal HO-1 expression since these animals were also used as control animals for paralleled studies about the role of microglial HO-1. As *Hmox1^fl/fl^* and *CD36*^−/−^ mice share the same C57BL/6 background, *Hmox1^fl/fl^* mice and microglia were utilized as controls for all in vivo and in vitro investigations to conserve animals.

Primary microglia (PMG) were isolated from *CD36^−/−^* or *Hmox1^fl/fl^* mice at P5 to P7 (decapitation) using enzymatic neural dissociation (Papain Neural Dissociation Kit; Miltenyi Biotec, Bergisch Gladbach, Germany) and in vitro mixed glia culture. Mouse brains were enzymatically dissociated according to the manufacturer’s instructions (cells from 3 brains pooled per plating). The resulting mixed glia culture containing astrocytes and microglia was cultivated in DMEM containing 1% penicillin–streptomycin, 10% FBS and M-CSF (10 ng/mL) in a humidified atmosphere with 5% CO_2_. After 1 week of cultivation, microglia were collected by shaking the cell culture plates at 200 rpm for 30 min every 2 to 3 days. Floating microglia were then collected from the supernatant and seeded onto 6-well plates for experiments at a density of 2 × 10^5^ cells per well. Microglial phenotype was confirmed by CD11b staining and flow cytometry.

BV-2 microglia cells (RRID:CVCL_0182) were cultured in DMEM containing 1% penicillin–streptomycin and 10% FBS in a humidified atmosphere with 5% CO_2_. Cells were seeded into 6-well plates at a density of 200,000 cells for individual experiments.

### 4.2. Circadian-Dependent Blood and CO Gas Treatment of Cells in Culture

**Figure 7:**
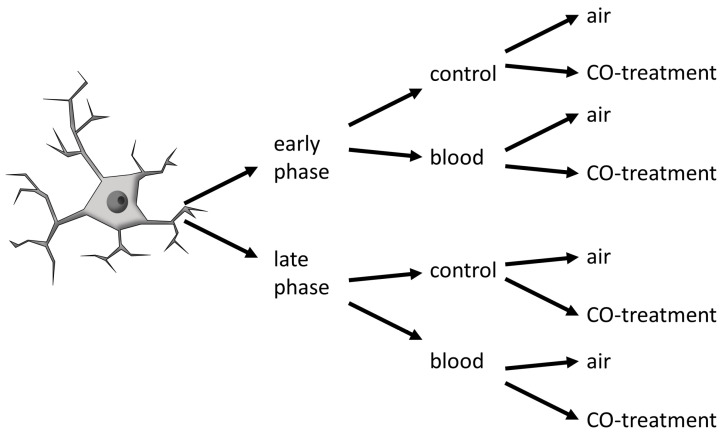
Experimental scheme of the in vitro PMG experiments; CO: carbon monoxide.

To obtain microglia cells in the early or late phases of the circadian timing, medium changes were performed at different time points throughout the day. The feasibility of circadian studies in vitro has been demonstrated previously showing robust in vitro expression of genes relevant to circadian control and the ability to induce these genes through various stimuli [54]. Circadian rhythms can be restored with suitable entrainment stimuli such as medium changes [54]. Therefore, experiments were performed 2, 8, 12 and 24 h after the medium change. Based on the measured gene expression of *Per-2* and *Cry-1*, early and late phases were defined in cells 2 h and 12 h after medium change. In Figure 7, the experimental scheme is represented with the treatments, which are further explained in the following.

For erythrophagocytosis experiments, 100 μL of blood was drawn from the mandibular vein of a C57BL/6 wild type animal, washed with PBS, and stained with a 356 μM amine-reactive, pH-sensitive fluorescent ester dye (pHrodo Green STP Ester; Thermo Fisher Scientific, Waltham, MA, USA) in 1 mL PBS for 30 min. PMG either in the early or late phase were then incubated with a 100 μL labeled red blood cell suspension for 1 h.

Cells designated for CO exposure were transferred on their plate to an air-tight, humidified chamber (C-Chamber; Biospherix, Parish, NY, USA) after initiating the red blood cell phagocytosis assay and treated with 250 ppm CO, 21% O_2_ and 5% CO_2_ for 1 h, which was controlled by an automated gas delivery system (Oxycycler; Biospherix, Parish, NY, USA).

### 4.3. Gene Expression Analyses from Cells and Tissue Samples

Cells were harvested by in-well lysis using the Trizol reagent. Tissue samples were immediately homogenized in Trizol and further processed for RNA purification. RNA concentration and purification were performed using spin columns (RNEasy mini kit, Qiagen, Hilden, Germany). cDNA libraries were acquired by reverse transcription (iScript cDNA synthesis kit, Bio-Rad, Feldkirchen, Germany). Then, gene expression of circadian rhythm genes was assessed via real-time PCR (SYBR green master mix, Applied Biosystems, Life Technologies GmbH, Darmstadt, Germany) with Rplp0 as the reference gene.

Primer sequences are:

1. *Per-2*

Forward: AGGATGTGGCAGGTAACAGG

Reverse: CGTAAGGGAACACACTGAGAGG

2. *Cry-1*

Forward: TGAGAAATATGGCGTTCCTTCC

Reverse: GTAAGTGCCTCAGTTTCTCCTC

3. *Rplp0*

Forward: GAGGAATCAGATGAGGATATGGGA

Reverse: AAGCAGGCTGACTTGGTTGC

4. *Per-2* (for siRNA experiments)

Forward: CACGAGCGGCTGCAGTAGTGA

Reverse: CGCTCCGCAGGGCATACTTC

### 4.4. RNA Interference

RNA interference in BV-2 cells was performed using Lipofectamine (Invitrogen, Life Technologies GmbH, Darmstadt, Germany) according to the manufacturer’s instructions. Cells were transfected either with siRNA specifically targeting *Per-2* (Silencer™ Select anti-Per2 Invitrogen, Life Technologies GmbH, Darmstadt, Germany, 4390771, s71487) or negative control siRNA (Silencer™ Select negative control No. 1 siRNA, Invitrogen, Life Technologies GmbH, Darmstadt, Germany, 4390843) (50 nM for 24 h). Subsequent downstream analyses, such as qPCR and FACS analysis, were conducted as indicated.

### 4.5. Flow Cytometry

Cells were harvested using a cell lifter, washed with flow cytometry buffer (PBS with 1% BSA, 2 mM EDTA, 0.05% Na-Azide) and stained with a PE-Cy7-tagged CD11b antibody (BioLegend, San Diego, CA, USA, Cat# 101216, RRID:AB_312799, 1:100, 30 min) and an Alexa Fluor 488-tagged antibody against CX3CR1 ((BioLegend, San Diego, CA, USA, Cat# 149021, RRID:AB_2565704), 1:100, 30 min). For experiments analyzing CD36 expression, microglia cells were harvested with a cell lifter, washed with flow cytometry buffer (PBS with 1% BSA, 2 mM EDTA, 0.05% Na-Azide) and stained with a PE-Cy7-tagged CD11b antibody (Biolegend, San Diego, CA, USA, 1:100, 30 min) and Alexa-488-tagged CD36 antibody (Biolegend, San Diego, CA, USA, 1:100, 30 min). The fluorescent signal intensity due to CD36 staining was evaluated in CD11b-positive cells. Flow cytometry was performed on a FACS LSR Frida (Becton Dickinson, Heidelberg, Germany). The relative percentage and mean fluorescence were calculated using FlowJo (version 10.9.0).

### 4.6. Western Blot Analysis

Cells were washed with PBS and lysed in radioimmunoprecipitation assay (RIPA) buffer supplemented with PhosStop (Sigma Aldrich, Taufkirchen, Germany, 4906845001) and protease inhibitor (Sigma Aldrich, Taufkirchen, Germany, 4693116001) and shaken at 1000 rpm for 10 min at 4 °C. Equal protein amounts were separated on a 10% SDS (Sigma Aldrich, Taufkirchen, Germany, R0278) polyacrylamide gel (TGX Stain-Free^TM^ FastCast^TM^ 161-0183 Bio-Rad, Feldkirchen, Germany) and transferred to a polyvinylidene difluoride (PVDF) membrane (Trans Blot Turbo Transfer Pack 1704157 Bio-Rad, Feldkirchen, Germany) using a Trans Blot Turbo Transfer System (Bio-Rad, Feldkirchen, Germany). TGX Stain-Free^TM^ FastCast^TM^ enables the visualization of the entire protein loaded on the gel, which is subsequently transferred to the membrane stain-free through UV light exposure. Consequently, membrane images were captured under UV light to display total protein. The cumulative total protein loaded as a sum of the total signal per lane under UV light served as reference and for normalization in the comparison of the expression levels of the target protein. Membranes were blocked with 5% skim milk BSA in Tween-20/TBS and incubated in the recommended dilution of specific antibodies (HO-1:Abcam, Cambridge, UK, Cat# ab52947, RRID:AB_880536, 1:1000; CD36: Abcam, Cambridge, UK, Cat# ab252922, RRID:AB_2922821, 1:1000) overnight at 4 °C. Then, membranes were incubated with the corresponding secondary antibody 1:5000 for chemiluminescence detection and developed on a Fusion FX imaging system with Western Lightning Plus-ECL (Perkin Elmer, Waltham, MA, USA, NEL103001EA). Total protein staining was used for normalization.

### 4.7. Co-Cultivation of PMG and HT22 for Viability Assay

PMG from *Hmox1^fl/fl^* or *CD36^−/−^* mice were seeded onto 24-well cell culture plates (Greiner, Kremsmünster, Austria) with a density of 20–30%. Hippocampal murine neuronal HT22 cells (RRID:CVCL_0321) were cultured in DMEM containing 1% penicillin–streptomycin and 10% FBS under a humidified atmosphere with 5% CO_2_. Cells were seeded into Thincert Cellculture Inserts with pores of 0.4 µm (Greiner, Kremsmünster, Austria) at a density of 2700 cells two days beforehand for individual experiments. The PMG cells’ medium was exchanged either 2 or 12 h before co-culturing and the addition of blood. Blood drawn from the mandibular vein of a C57BL/6 wild type animal was washed with PBS and diluted in the culture medium. Per each 24-well, 10 µL of blood was added to the microglia culture. Inserts were immediately placed into the PMG wells and incubated for 12 h at 37 °C with 5% CO_2_ in a humidified atmosphere. After 11 h, the CO-treated group was incubated for 1 h with 250 ppm CO, 21% O2 and 5% CO_2_. The viability of the HT22 cells was measured using the CellTiter 96^®^ AQueous One Solution Cell Proliferation Assay (MTS by Promega, Madison, WI, USA) according to the manufacturer’s protocol in a plate reader (Tecan, Männerdorf, Switzerland, Infinity 200 PRO). For this, inserts were removed from the co-culture, the medium was aspirated and a 1/6 diluted MTS solution was added to the inserts. After 2 h of incubation at 37 °C and 5% CO_2_ in a humidified atmosphere, 80 µL of the incubated MTS solution was transferred into a 96-well plate for spectral analysis using a plate reader.

### 4.8. Animal Care and Anesthesia

Animals were provided with a standard rodent diet ad libitum and maintained on a 12 h light/12 h dark cycle in cages accommodating 1–5 mice. For in vivo experiments, male mice were used, and for in vitro study, male and female mice were used. All surgical procedures and manipulations were conducted under general anesthesia using ketamine (100 mg/kg) and xylazine (5 mg/kg) (administrated via intraperitoneal injection) and body temperature maintenance. For surgical procedures, buprenorphine (50 µg/kg) was applied subcutaneously to treat potential pain. Euthanasia of anesthetized mice took place through trans-cardiac perfusion.

### 4.9. SAH Stroke Model

SAH was achieved by injection of blood. Following anesthesia induction, the head was fixed in a stereotactic mounting. A midline skin incision exposed the anterior skull. A 0.8 mm burr hole was drilled 3.5 mm anterior to the bregma, into the skull at a caudal angle of 40°. A total of 40 µL of blood was withdrawn from a blood donor. A 27-G needle was slowly advanced through the burr hole at a 40° angle until the skull at the base of the brain was reached. Then, the blood was slowly injected through the 27-G needle. The needle remained in place for 1 min to prevent backflow. After confirming no overt bleeding, the skin was sutured and animals were allowed to recover from anesthesia under close supervision, maintaining body temperature with infrared light. The mice were treated with buprenorphine (50 µg/kg) three times a day for the initial three days postoperatively.

SAH was either induced at zeitgeber time (ZT) 2 or 12, corresponding to the end of the 2nd and the 12th hour of the 12 h light cycle (with the light cycle starting at 6 o’clock am and ending at 6 o’clock pm; ZT2 = 8 o’clock am; ZT12 = 6 o’clock pm). The selection of ZT2 and ZT12 as the time points of SAH induction aligns with the nadir and peak within the circadian gene expression profile.

### 4.10. CO Gas Treatment of Mice

Following SAH, animals were randomly assigned to receive either synthetic air for 1 h or CO treatment in a custom-made chamber. Animals had unrestricted access to food and water during the treatment. Pre-mixed air with 250 ppm CO was used. CO concentration was continuously monitored throughout the exposure with an infrared gas analyzer. Treatment commenced immediately after the SAH and recurred every 24 h for 1 to 7 days, depending on the specified readouts.

### 4.11. Hematoxylin/Eosin Staining and Evaluation of Cerebral Vasospasm

Seven days after SAH, animals were deeply anesthetized with ketamine and xylazine and transcardially perfused with TBS followed by 4% PFA. Brains were extracted and fixed in 4% PFA for 18 h, cryoprotected in sucrose and then frozen for sectioning into 8 μm slices. Hematoxylin and Eosin staining were applied and digital representative images of 3 consecutive cross-sections of middle cerebral arteries (MCA) per animal were obtained. The lumen radius/wall thickness ratio was quantified using Image J (version 1.47v) to assess vasospasm.

### 4.12. Immunohistochemistry

Frozen brains were cut into 8 µm serial coronal sections. The glass slides with brain sections were heated in 1× citrate buffer pH 6 (Zytomed System GmbH, Berlin, Germany, K035) in a microwave (3 × 5 min 800 W). After a 20 min cool-down, permeabilization was carried out with 0.2% Tween/PBS (Ter-119 with Iba-1) or 0.5% Triton X100/PBS (TMEM119 with Iba-1) for 10 min at room temperature. Slides were then blocked in BSA/PBS for 30 min (4% BSA for Ter119 with Iba-1; 10% BSA for TMEM119 with Iba-1at room temperature). Staining was performed with primary antibodies against Iba-1 (Abcam, Cambridge, UK, Cat# ab5076, RRID:AB_2224402; 1:300), TMEM119 (Abcam, Cambridge, UK, Cat# ab209064, RRID:AB_2800343; 1:300) or TER-119 (Abcam, Cambridge, UK, Cat# ab91113, RRID:AB_2050384; 1:100) at 4 °C overnight. For fluorescent imaging, sections were incubated with the corresponding secondary antibody for 1 h (for Iba-1 anti-goat Alexa Fluor 488, Abcam, Cambridge, UK, Cat# ab150129, RRID:AB_2687506 or anti-goat Alexa Fluor 555, Abcam, Cambridge, UK, Cat# ab150130, RRID:AB_2927775; for TER-119 anti-rat Alexa Fluor 555, Abcam, Cambridge, UK, Cat# ab150154, RRID:AB_2813834; 1:500; for TMEM119 anti-rabbit Alexa Fluor 555, Abcam, Cambridge, UK, Cat# ab150073, RRID:AB_2636877). Nuclear counterstaining was performed with DAPI (4′,6-diamidino-2-phenylindole) (5 min), and cover slides were added together with mounting medium (Agilent, Santa Clara, CA, USA, Dako, S302380-2). Slides were examined under a bright field and fluorescence microscope (Zeiss, Oberkochen, Germany, AxioObserver Invert). To ensure consistency in cell quantification, three to four images were obtained from each area of interest for three mice per group, resulting in 9–12 analyzed images per group. Iba-1 and TMEM119 were utilized as microglia markers. For the analysis of microglia phagocytosing erythrocytes, cells with co-localization of TER-119 and Iba-1 were counted. Activated microglia are characterized by larger cell bodies with retracted extensions, while resting microglia show smaller cell bodies with extended ramifications. Therefore, we measured the soma size by area of Iba-1-positive microglia as an activation hallmark using ZEN (blue edition version 2.5) software from Carl Zeiss Microscopy, Oberkochen, Germany.

### 4.13. Barnes Maze Behavioral Studies

**Figure 8:**
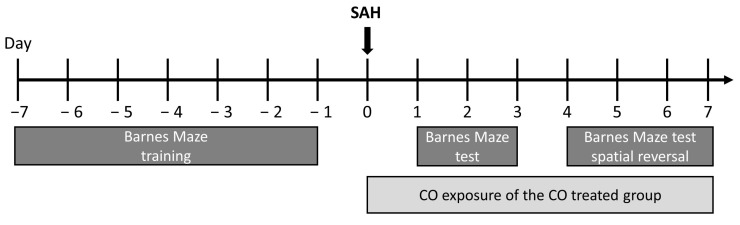
Experimental scheme of the in vivo mouse experiments; SAH: subarachnoidal hemorrhage; CO: carbon monoxide.

Spatial learning was assessed using the Barnes maze, consisting of a white circular platform with a diameter of 120 cm and 40 equal-sized holes along the perimeter. Below one hole, a box to which the animals could escape to from the open platform was placed. As an aversive stimulus to exit the platform, a bright light source was placed above the setup. A rewarding stimulus was implemented by placing food pellets into the escape box. Cardboard symbols with distinct visual cues were placed around the platform. During the initial acquisition period, the location of the escape box and the visual cues remained constant. Mice underwent initial spatial acquisition for 7 days, with three daily trials per animal, allowing 3 min of exploration on the maze per animal per trail. Mice were allowed to remain in the box for 30 s once they had entered the escape box. The inter-trial interval was 30 min for each animal, with a thorough cleaning of the platform after each animal to eliminate olfactory cues. Animals who failed to enter the escape box within 3 min were gently guided to the correct hole and into the box. Cognitive competence was assessed based on the total latency (time taken to find the goal box) and the error number (number of holes explored before finding the goal box). SAH was induced after 7 days of training (Figure 8). Spatial memory testing involved one trial per animal per day from day one to day seven after SAH. To test for flexibility and relearning, the location of the goal box was changed to the exact opposite position on day four (spatial reversal) with visual cues kept constant at the initial position. Maze procedures were performed by an investigator blinded to the genotype and treatment groups.

### 4.14. Statistics

Sample size calculations for in vivo experiments were conducted using G Power 3.1. The primary endpoint for calculating group size was the neurocognitive function (latency (sec)) during the spatial reversal phase (day 4–7, 4 measurement points respective genotype, SAH at ZT2 and ZT12 ± CO). The primary endpoint “neurocognitive function” was selected based on the greatest translational relevance and was used as the basis for the statistical calculations of the group size. Neuronal apoptosis analyzed by TUNEL staining in brain slices after 7 days was used for secondary endpoint. Repeated measures ANOVA was employed as a biometric test. The expected effect size was determined from published preliminary tests [13] and current projects: partial η2 = 0.4; α = 0.05, β = 0.8, resulting in a group size of *n* = 8. Sample size determination for downstream in vitro analyses relied on our previously published studies with microglia and CO [12,14].

Data were analyzed using the statistical software GraphPad Prism Version 10. Results are presented as box plots (whiskers indicate minimum and maximum; the line in the box marks the median; the “+” in the box shows the position of the mean). For all data, tests for normal distribution (D’Agostino and Pearson test; Shapiro–Wilk test) were conducted. These results were used to determine whether to employ parametric or non-parametric tests. Two groups were compared using Student’s *t*-test or for not normally distributed results using Mann–Whitney test. For relative comparisons versus an averaged control group, one-column statistics with one sample *t*-tests or Wilcoxon test were used. Normalization of flow cytometric data to the daily control was necessary due to common fluctuations in the baseline. Microscopic data were normalized as the selected sections differ in general cell counts. The statistical tests were performed two-tailed. A *p*-value smaller than 0.05 was considered to be statistically significant.

### 4.15. Study Approval

All procedures involving the animals received approval from the Committee of Animal Care at the University of Freiburg (Permit No. G18/17 and X18-04F) and were conducted in accordance with the ARRIVE guidelines and the Directive 2010/63/EU by the European Union.

## Data Availability

The data that support the findings of this study are available from the corresponding author upon reasonable request.

## References

[1] Temes R.E., Bleck T., Dugar S., Ouyang B., Mohammad Y., John S., Patel P., Lee V., Prabhakaran S., Quigg M. (2012). Circadian variation in ictus of aneurysmal subarachnoid hemorrhage. Neurocrit. Care.

[2] Chieregato A., Tagliaferri F., Cocciolo F., Boari B., Gallerani M., Manfredini R. (2007). Can circadian rhythms influence onset and outcome of nontraumatic subarachnoid hemorrhage?. Am. J. Emerg. Med..

[3] Manfredini R., Boari B., Smolensky M.H., Salmi R., la Cecilia O., Maria Malagoni A., Haus E., Manfredini F. (2005). Circadian variation in stroke onset: Identical temporal pattern in ischemic and hemorrhagic events. Chronobiol. Int..

[4] Karatsoreos I.N., Bhagat S., Bloss E.B., Morrison J.H., McEwen B.S. (2011). Disruption of circadian clocks has ramifications for metabolism, brain, and behavior. Proc. Natl. Acad. Sci. USA.

[5] Kolbe I., Leinweber B., Brandenburger M., Oster H. (2019). Circadian clock network desynchrony promotes weight gain and alters glucose homeostasis in mice. Mol. Metab..

[6] Shearman L.P., Sriram S., Weaver D.R., Maywood E.S., Chaves I., Zheng B., Kume K., Lee C.C., van der Horst G.T., Hastings M.H. (2000). Interacting molecular loops in the mammalian circadian clock. Science.

[7] Panda S., Antoch M.P., Miller B.H., Su A.I., Schook A.B., Straume M., Schultz P.G., Kay S.A., Takahashi J.S., Hogenesch J.B. (2002). Coordinated transcription of key pathways in the mouse by the circadian clock. Cell.

[8] Zhang R., Lahens N.F., Ballance H.I., Hughes M.E., Hogenesch J.B. (2014). A circadian gene expression atlas in mammals: Implications for biology and medicine. Proc. Natl. Acad. Sci. USA.

[9] Eckle T., Hartmann K., Bonney S., Reithel S., Mittelbronn M., Walker L.A., Lowes B.D., Han J., Borchers C.H., Buttrick P.M. (2012). Adora2b-elicited Per2 stabilization promotes a HIF-dependent metabolic switch crucial for myocardial adaptation to ischemia. Nat. Med..

[10] Chen P., Li C., Pang W., Zhao Y., Dong W., Wang S., Zhang J. (2009). The protective role of Per2 against carbon tetrachloride-induced hepatotoxicity. Am. J. Pathol..

[11] Wiebking N., Maronde E., Rami A. (2013). Increased neuronal injury in clock gene Per-1 deficient-mice after cerebral ischemia. Curr. Neurovasc. Res..

[12] Schallner N., Lieberum J.L., Gallo D., LeBlanc R.H., Fuller P.M., Hanafy K.A., Otterbein L.E. (2017). Carbon Monoxide Preserves Circadian Rhythm to Reduce the Severity of Subarachnoid Hemorrhage in Mice. Stroke A J. Cereb. Circ..

[13] Schallner N., Pandit R., LeBlanc R., Thomas A.J., Ogilvy C.S., Zuckerbraun B.S., Gallo D., Otterbein L.E., Hanafy K.A. (2015). Microglia regulate blood clearance in subarachnoid hemorrhage by heme oxygenase-1. J. Clin. Investig..

[14] Kaiser S., Selzner L., Weber J., Schallner N. (2020). Carbon monoxide controls microglial erythrophagocytosis by regulating CD36 surface expression to reduce the severity of hemorrhagic injury. Glia.

[15] McGilvray I.D., Serghides L., Kapus A., Rotstein O.D., Kain K.C. (2000). Nonopsonic monocyte/macrophage phagocytosis of Plasmodium falciparum-parasitized erythrocytes: A role for CD36 in malarial clearance. Blood.

[16] Fang H., Chen J., Lin S., Wang P., Wang Y., Xiong X., Yang Q. (2014). CD36-mediated hematoma absorption following intracerebral hemorrhage: Negative regulation by TLR4 signaling. J. Immunol..

[17] Flores J.J., Klebe D., Rolland W.B., Lekic T., Krafft P.R., Zhang J.H. (2016). PPARgamma-induced upregulation of CD36 enhances hematoma resolution and attenuates long-term neurological deficits after germinal matrix hemorrhage in neonatal rats. Neurobiol. Dis..

[18] Mu Q., Wang L., Hang H., Liu C., Wu G. (2017). Rosiglitazone pretreatment influences thrombin-induced phagocytosis by rat microglia via activating PPARgamma and CD36. Neurosci. Lett..

[19] Dioum E.M., Rutter J., Tuckerman J.R., Gonzalez G., Gilles-Gonzalez M.A., McKnight S.L. (2002). NPAS2: A gas-responsive transcription factor. Science.

[20] Lukat-Rodgers G.S., Correia C., Botuyan M.V., Mer G., Rodgers K.R. (2010). Heme-based sensing by the mammalian circadian protein CLOCK. Inorg. Chem..

[21] Chaurasia S.S., Pozdeyev N., Haque R., Visser A., Ivanova T.N., Iuvone P.M. (2006). Circadian clockwork machinery in neural retina: Evidence for the presence of functional clock components in photoreceptor-enriched chick retinal cell cultures. Mol. Vis..

[22] Soares N.L., Paiva I., Bravo J., Queiroga C.S.F., Melo B.F., Conde S.V., Romao C.C., Summavielle T., Vieira H.L.A. (2022). Carbon Monoxide Modulation of Microglia-Neuron Communication: Anti-Neuroinflammatory and Neurotrophic Role. Mol. Neurobiol..

[23] Kamat P.K., Ahmad A.S., Dore S. (2019). Carbon monoxide attenuates vasospasm and improves neurobehavioral function after subarachnoid hemorrhage. Arch Biochem. Biophys.

[24] Czeisler C.A., Duffy J.F., Shanahan T.L., Brown E.N., Mitchell J.F., Rimmer D.W., Ronda J.M., Silva E.J., Allan J.S., Emens J.S. (1999). Stability, precision, and near-24-hour period of the human circadian pacemaker. Science.

[25] Frase S., Kaiser S., Steimer M., Selzner L., Foit N.A., Niesen W.D., Schallner N. (2021). Patients with Subarachnoid Hemorrhage Exhibit Disturbed Expression Patterns of the Circadian Rhythm Gene Period-2. Life.

[26] Suzuki H., Kawakita F., Asada R. (2022). Neuroelectric Mechanisms of Delayed Cerebral Ischemia after Aneurysmal Subarachnoid Hemorrhage. Int. J. Mol. Sci..

[27] Chen J., Li M., Liu Z., Wang Y., Xiong K. (2022). Molecular mechanisms of neuronal death in brain injury after subarachnoid hemorrhage. Front. Cell. Neurosci..

[28] Solar P., Zamani A., Lakatosova K., Joukal M. (2022). The blood-brain barrier and the neurovascular unit in subarachnoid hemorrhage: Molecular events and potential treatments. Fluids Barriers CNS.

[29] Zhang Y., Zhang T., Li Y., Guo Y., Liu B., Tian Y., Wu P., Shi H. (2022). Metformin attenuates early brain injury after subarachnoid hemorrhage in rats via AMPK-dependent mitophagy. Exp. Neurol..

[30] Li R., Zhao M., Yao D., Zhou X., Lenahan C., Wang L., Ou Y., He Y. (2022). The role of the astrocyte in subarachnoid hemorrhage and its therapeutic implications. Front. Immunol..

[31] Matz P., Turner C., Weinstein P.R., Massa S.M., Panter S.S., Sharp F.R. (1996). Heme-oxygenase-1 induction in glia throughout rat brain following experimental subarachnoid hemorrhage. Brain Res..

[32] Sutherland B.A., Rahman R.M., Clarkson A.N., Shaw O.M., Nair S.M., Appleton I. (2009). Cerebral heme oxygenase 1 and 2 spatial distribution is modulated following injury from hypoxia-ischemia and middle cerebral artery occlusion in rats. Neurosci. Res..

[33] Takeda A., Kimpara T., Onodera H., Itoyama Y., Shibahara S., Kogure K. (1996). Regional difference in induction of heme oxygenase-1 protein following rat transient forebrain ischemia. Neurosci. Lett..

[34] Turner C.P., Panter S.S., Sharp F.R. (1999). Anti-oxidants prevent focal rat brain injury as assessed by induction of heat shock proteins (HSP70, HO-1/HSP32, HSP47) following subarachnoid injections of lysed blood. Brain Res. Mol. Brain Res..

[35] Shimada Y., Tsunoda H., Zang L., Hirano M., Oka T., Tanaka T. (2009). Synergistic induction of heme oxygenase-1 by nicaraven after subarachnoid hemorrhage to prevent delayed cerebral vasospasm. Eur. J. Pharmacol..

[36] Zhang F., Wang S., Zhang M., Weng Z., Li P., Gan Y., Zhang L., Cao G., Gao Y., Leak R.K. (2012). Pharmacological induction of heme oxygenase-1 by a triterpenoid protects neurons against ischemic injury. Stroke.

[37] Saleem S., Zhuang H., Biswal S., Christen Y., Dore S. (2008). Ginkgo biloba extract neuroprotective action is dependent on heme oxygenase 1 in ischemic reperfusion brain injury. Stroke.

[38] Wang H.Q., Xu Y.X., Zhu C.Q. (2012). Upregulation of heme oxygenase-1 by acteoside through ERK and PI3 K/Akt pathway confer neuroprotection against beta-amyloid-induced neurotoxicity. Neurotox. Res..

[39] Chen G., Fang Q., Zhang J., Zhou D., Wang Z. (2011). Role of the Nrf2-ARE pathway in early brain injury after experimental subarachnoid hemorrhage. J. Neurosci. Res..

[40] Foresti R., Bains S.K., Pitchumony T.S., de Castro Bras L.E., Drago F., Dubois-Rande J.L., Bucolo C., Motterlini R. (2013). Small molecule activators of the Nrf2-HO-1 antioxidant axis modulate heme metabolism and inflammation in BV2 microglia cells. Pharmacol. Res..

[41] Li F., Faustino J., Woo M.S., Derugin N., Vexler Z.S. (2015). Lack of the scavenger receptor CD36 alters microglial phenotypes after neonatal stroke. J. Neurochem..

[42] Li X., Melief E., Postupna N., Montine K.S., Keene C.D., Montine T.J. (2015). Prostaglandin E2 Receptor Subtype 2 Regulation of Scavenger Receptor CD36 Modulates Microglial Abeta42 Phagocytosis. Am. J. Pathol..

[43] Yamanaka M., Ishikawa T., Griep A., Axt D., Kummer M.P., Heneka M.T. (2012). PPARgamma/RXRalpha-induced and CD36-mediated microglial amyloid-beta phagocytosis results in cognitive improvement in amyloid precursor protein/presenilin 1 mice. J. Neurosci..

[44] Fadok V.A., Warner M.L., Bratton D.L., Henson P.M. (1998). CD36 is required for phagocytosis of apoptotic cells by human macrophages that use either a phosphatidylserine receptor or the vitronectin receptor (alpha v beta 3). J. Immunol..

[45] Hajri T., Han X.X., Bonen A., Abumrad N.A. (2002). Defective fatty acid uptake modulates insulin responsiveness and metabolic responses to diet in CD36-null mice. J. Clin. Investig..

[46] Cifarelli V., Appak-Baskoy S., Peche V.S., Kluzak A., Shew T., Narendran R., Pietka K.M., Cella M., Walls C.W., Czepielewski R. (2021). Visceral obesity and insulin resistance associate with CD36 deletion in lymphatic endothelial cells. Nat. Commun..

[47] Mistry J.J., Hellmich C., Moore J.A., Jibril A., Macaulay I., Moreno-Gonzalez M., Di Palm F., Beraza N., Bowles K.M., Rushworth S.A. (2021). Free fatty-acid transport via CD36 drives beta-oxidation-mediated hematopoietic stem cell response to infection. Nat. Commun..

[48] Van Dycke K.C., Pennings J.L., van Oostrom C.T., van Kerkhof L.W., van Steeg H., van der Horst G.T., Rodenburg W. (2015). Biomarkers for circadian rhythm disruption independent of time of day. PLoS ONE.

[49] Pan X., Queiroz J., Hussain M.M. (2020). Nonalcoholic fatty liver disease in CLOCK mutant mice. J. Clin. Investg..

[50] Prabhat A., Malik I., Jha N.A., Bhardwaj S.K., Kumar V. (2020). Developmental effects of constant light on circadian behaviour and gene expressions in zebra finches: Insights into mechanisms of metabolic adaptation to aperiodic environment in diurnal animals. J. Photochem. Photobiol. B.

[51] Xu W., Xu R., Li X., Zhang H., Wang X., Zhu J. (2016). Downregulating hypoxia-inducible factor-1alpha expression with perfluorooctyl-bromide nanoparticles reduces early brain injury following experimental subarachnoid hemorrhage in rats. Am. J. Transl. Res..

[52] Jin J., Duan J., Du L., Xing W., Peng X., Zhao Q. (2022). Inflammation and immune cell abnormalities in intracranial aneurysm subarachnoid hemorrhage (SAH): Relevant signaling pathways and therapeutic strategies. Front. Immunol..

[53] Peliciari-Garcia R.A., Bargi-Souza P., Young M.E., Nunes M.T. (2018). Repercussions of hypo and hyperthyroidism on the heart circadian clock. Chronobiol. Int..

[54] Prolo L.M., Takahashi J.S., Herzog E.D. (2005). Circadian rhythm generation and entrainment in astrocytes. J. Neurosci..

[55] Otterbein L.E., Bach F.H., Alam J., Soares M., Tao Lu H., Wysk M., Davis R.J., Flavell R.A., Choi A.M. (2000). Carbon monoxide has anti-inflammatory effects involving the mitogen-activated protein kinase pathway. Nat. Med..

[56] Stupfel M., Bouley G. (1970). Physiological and biochemical effects on rats and mice exposed to small concentrations of carbon monoxide for long periods. Ann. N. Y. Acad. Sci..

[57] D’Amico G., Lam F., Hagen T., Moncada S. (2006). Inhibition of cellular respiration by endogenously produced carbon monoxide. J. Cell Sci..

